# Practical strategies for achieving system change in the US: lessons and insights from the CONQUEST quality improvement programme

**DOI:** 10.1017/S1463423625100170

**Published:** 2025-06-23

**Authors:** Alexander Evans, Jill VanWyk, Margee Kerr, Amy Couper, Wilson D. Pace, Yasir Tarabichi, Rachel Pullen, Michael Pollack, M. Bradley Drummond, Jill Ohar, Catherine Meldrum, MeiLan K. Han, Alan Kaplan, Tonya Winders, Juan Wisnivesky, Barry Make, Alex Federman, Victoria Carter, Katie Lang, Douglas Mapel, Nicola A. Hanania, Daiana Stolz, Fernando J. Martinez, David Price

**Affiliations:** 1 Observational and Pragmatic Research Institute Pte Ltd, Singapore, Singapore; 2 Department of Family Medicine, University of Colorado School of Medicine, Aurora, CO, USA; 3 Optimum Patient Care Global, Cambridge, UK; 4 DARTNet Institute, Aurora, CO, USA; 5 University of Colorado, Denver, CO, USA; 6 Pulmonologist at Metro Health Medical Center, Cleveland, OH, USA; 7 BioPharmaceuticals Medical, AstraZeneca, Wilmington, DE, USA; 8 Division of Pulmonary Diseases and Critical Care Medicine, University of North Carolina at Chapel Hill, Chapel Hill, NC, USA; 9 Department of Internal Medicine, Wake Forest University, Winston-Salem, NC, USA; 10 Pulmonary and Critical Care Medicine Division, Department of Internal Medicine, University of Michigan Health System, Ann Arbor, MI, USA; 11 University of Michigan, Ann Arbor, MI, USA; 12 Family Physician Airways Group of Canada, Stouffville, ON, Canada; 13 University of Toronto, Toronto, Canada; 14 Global Allergy & Airways Patient Platform, Vienna, Austria; 15 Icahn School of Medicine at Mount Sinai, New York, NY, USA; 16 Department of Medicine, National Jewish Health, Denver, CO, USA; 17 Division of General Internal Medicine, Icahn School of Medicine, New York, NY, USA; 18 University of New Mexico College of Pharmacy, Albuquerque, NM, USA; 19 Section of Pulmonary and Critical Care Medicine, and Director of the Airways Clinical Research Center, Baylor College of Medicine, Houston, TX, USA; 20 Clinic of Respiratory Medicine and Faculty of Medicine, University of Freiburg, Freiburg, Germany; 21 University of Massachusetts Chan Medical School/UMassMemorial Health, Worcester, MA, USA; 22 Centre of Academic Primary Care, Division of Applied Health Sciences, University of Aberdeen, Aberdeen, UK

**Keywords:** COPD, implementation, integrated health care primary care, quality improvement

## Abstract

**Background:**

Quality improvement programmes (QIPs) are designed to enhance patient outcomes by systematically introducing evidence-based clinical practices. The CONQUEST QIP focuses on improving the identification and management of patients with COPD in primary care. The process of developing CONQUEST, recruiting, preparing systems for participation, and implementing the QIP across three integrated healthcare systems (IHSs) is examined to identify and share lessons learned.

**Approach and development:**

This review is organized into three stages: 1) development, 2) preparing IHSs for implementation, and 3) implementation. In each stage, key steps are described with the lessons learned and how they can inform others interested in developing QIPs designed to improve the care of patients with chronic conditions in primary care.

Stage 1 was establishing and working with steering committees to develop the QIP Quality Standards, define the target patient population, assess current management practices, and create a global operational protocol. Additionally, potential IHSs were assessed for feasibility of QIP integration into primary care practices. Factors assessed included a review of technological infrastructure, QI experience, and capacity for effective implementation.

Stage 2 was preparation for implementation. Key was enlisting clinical champions to advocate for the QIP, secure participation in primary care, and establish effective communication channels. Preparation for implementation required obtaining IHS approvals, ensuring Health Insurance Portability and Accountability Act compliance, and devising operational strategies for patient outreach and clinical decision support delivery.

Stage 3 was developing three IHS implementation models. With insight into the local context from local clinicians, implementation models were adapted to work with the resources and capacity of the IHSs while ensuring the delivery of essential elements of the programme.

**Conclusion:**

Developing and launching a QIP programme across primary care practices requires extensive groundwork, preparation, and committed local champions to assist in building an adaptable environment that encourages open communication and is receptive to feedback.

## Introduction

Chronic obstructive pulmonary disease (COPD) is a progressive lung disorder characterized by enduring respiratory symptoms, exacerbations, and airflow limitation (WHO, 2024; GOLD Report, 2024). This increased symptom burden affects patient health, often resulting in reduced quality of life (QoL), increased disability, and premature death (Sullivan *et al.*, 2018; Hurst *et al.*, 2020). The Centers for Disease Control and Prevention currently estimate COPD prevalence in the US to be 6.1% when standardized for age, representing approximately 14.2 million adults (Carlson, 2022). Patients with COPD frequently experience respiratory exacerbations that necessitate ongoing medical care, placing a substantial burden on healthcare systems (Bartels *et al.*, 2018; Anees ur Rehman *et al.*, 2020; Mannino *et al.*, 2024; Roberts *et al.*, 2024). Investigations into COPD healthcare utilization found it contributed significantly to all-cause and respiratory-specific hospitalizations, emergency department visits, and decreases in self-perceived health (Murphy *et al.*, 2017).

In the US, chronic conditions like COPD are predominantly managed in primary care, where primary care providers (PCPs) are often the first point of contact for patients and are essential in the early detection, diagnosis, and ongoing management of COPD (Criner and Han, 2018; Skolnik *et al.*, 2018). The enduring nature of COPD represents significant challenges to primary care, with constraints on the length of visits, limited resources (e.g., staff capacity), and complex network structures contributing to an increased difficulty in disease management (Han *et al.*, 2016). This is especially challenging when overseeing patients at higher risk, i.e., those who experience more frequent symptoms and exacerbations of COPD (Halpin *et al.*, 2023; Kerr *et al.*, 2023). This group of patients is not only at a higher risk of morbidity and mortality from COPD itself but also for other cardiopulmonary comorbidities (Kerkhof *et al.*, 2019; Daniels *et al.*, 2024; Singh *et al.*, 2024). Earlier identification of high-risk status among patients diagnosed and those who are undiagnosed with COPD despite experiencing health events consistent with COPD exacerbations, (e.g., lower respiratory tract infections) allows for timely intervention that can slow disease progression, improve QoL, and prevent severe exacerbations (Han *et al.*, 2015; Simmering *et al.*, 2016).

Currently, few quality improvement programmes (QIPs) are focusing on COPD management within primary care, despite numerous studies highlighting the space for such programmes to flourish (Halpin *et al.*, 2023; Kerr *et al.*, 2023). QIPs seek to systematically improve healthcare processes, outcomes, provider fulfilment, and patient satisfaction by implementing evidence-based practices (Batalden and Davidoff, 2007; Backhouse and Ogunlayi, 2020; Russ *et al.*, 2023). QIPs enhance the quality of care by improving early detection and treatment, standardizing care, optimizing resource use, and fostering continuous professional development (Agency for Healthcare and Quality, 2018; The Health Foundation, 2021). Given the burden COPD places on healthcare systems, there is a demonstrable opportunity for QIPs to increase efficiencies and reduce barriers in COPD identification and management (Gershon *et al.*, 2018; Iheanacho *et al.*, 2020). However, previous studies into QI implementation in chronic diseases highlight a variety of developmental, operational, and clinical challenges that create difficulties in ensuring effective, efficient, and sustained practice change within primary care (Dixon-Woods, McNicol and Martin, 2012; Kiran, Rozmovits and O’Brien, 2023; Carbonell *et al.*, 2024). A prior QIP across two health systems in the southeastern United States highlighted limited uptake at baseline of guideline-recommended diagnostic and therapeutic approaches; these improved with a limited intervention (Martinez *et al.*, 2021).

The COllaboratioN on QUality improvement initiative for achieving Excellence in STandards of COPD care (CONQUEST) was established in 2020 by Optimum Patient Care Global (OPC) and collaborators, as a global QIP with additional clinical decision support (CDS) focused on enhancing the care and outcomes for patients with COPD (Pullen *et al.*, 2021; Alves *et al.*, 2022). As its main objectives, CONQUEST aims to improve patient health outcomes by reducing COPD exacerbations, COPD-related hospital admissions/readmissions, and major adverse cardiopulmonary events (S-Table 1) (Alves *et al.*, 2022).

The efficacy of the CONQUEST QIP will be assessed through a cluster-randomized trial (PREVAIL US, registration number NCT05306743), and results will be presented upon study conclusion. However, we felt it important at this time to share insight from the development and implementation of CONQUEST. In this development paper, we outline the steps taken to build the CONQUEST QIP and describe the strategies adopted to identify, recruit, and prepare healthcare systems for participation. We then share insights from implementing this primary care-centred chronic disease programme in three US healthcare systems. In doing so, we aim to provide practical and actionable strategies for others interested in quality improvement implementation. This work contributes to the growing body of literature on QIPs and achieving system change across complex healthcare networks.

## Stage 1: development of the CONQUEST QIP and identifying suitable US healthcare systems

### Development of the CONQUEST QIP

**Figure 1.**
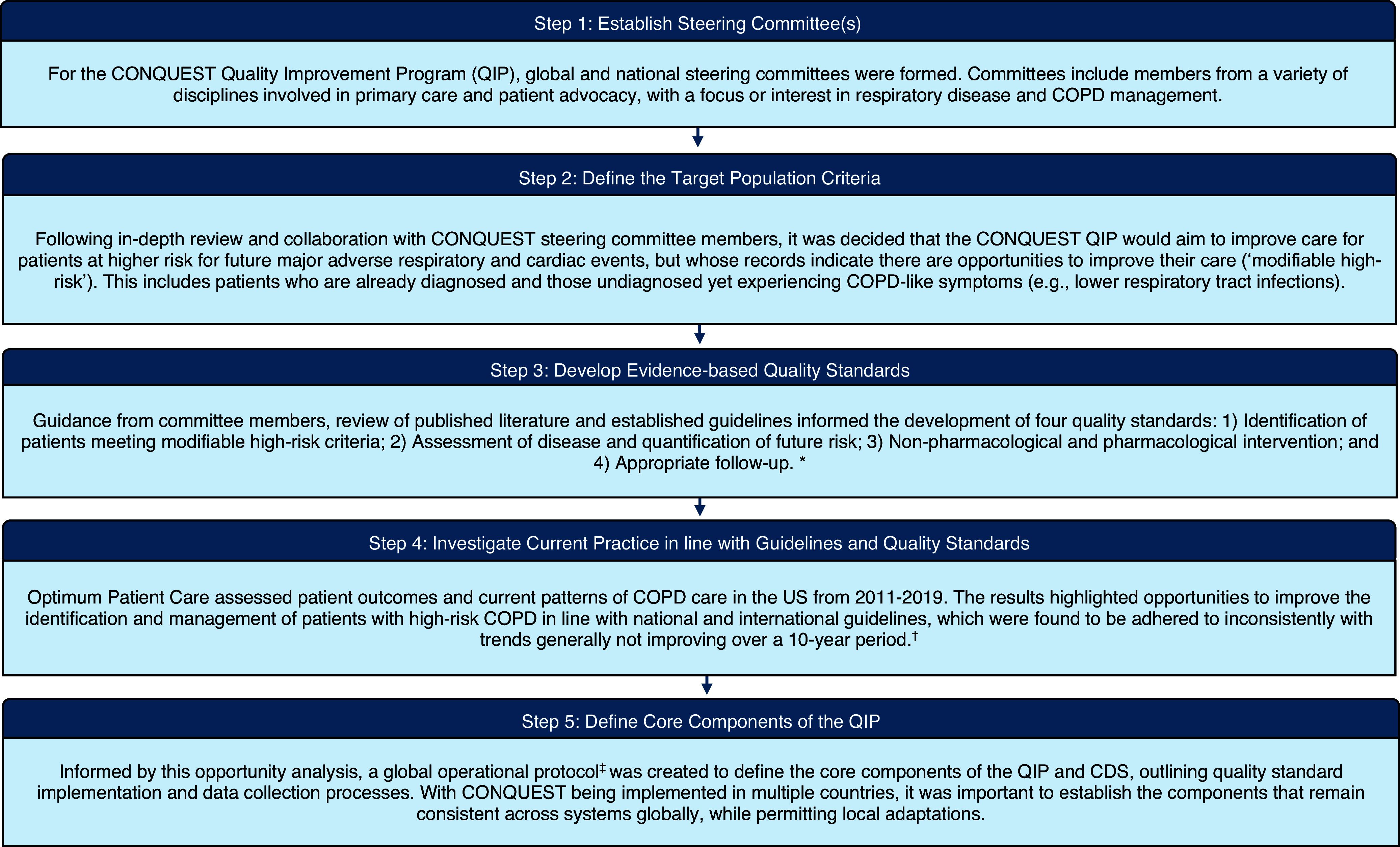
Flow chart of summarized steps to developing the CONQUEST quality improvement programme. Abbreviations: COPD, Chronic obstructive pulmonary disease; CONQUEST, COllaboratioN on QUality improvement initiative for achieving Excellence in STandards of COPD care; QIP, quality improvement programme. * Pullen *et al.*, 2021; † Kerr *et al.* 2023; ‡ Alves *et al.*, 2022.

Five key steps were taken in developing the CONQUEST QIP (Figure 1) to ensure that the programme was relevant, evidence-based, and informed by the latest expertise. Steps taken include the following: 1) establishing steering committees, 2) defining the patient population, 3) developing evidence-based quality standards (Pullen *et al.*, 2021), 4) assessing current practices against these standards (Kerr *et al.*, 2023), and 5) formalizing components of the QIP and CDS in a global operational protocol (GOP) (Alves *et al.*, 2022).

### Identifying suitable US healthcare systems

The CONQUEST QIP is designed to be adaptable to implementation in smaller practices and large primary care networks. However, for optimal success, certain prerequisites must be fulfilled. To determine whether implementation is possible, a minimum requirement checklist was developed and included in the CONQUEST GOP (Alves *et al.*, 2022). The checklist includes 19 requirements covering infrastructure, resources, clinical activity, and data analysis/sharing. For individual practices, it is a helpful tool to assess feasibility and identify areas requiring development prior to introducing CONQUEST.

Evaluating long-term outcomes among patients participating in CONQUEST is an important component of the QIP. As such, we aimed to implement CONQUEST across healthcare systems with diverse primary care networks to gain feedback, assess quality improvement (QI) impact, and modify accordingly. Integrated healthcare systems (IHSs), in which records of patient care from primary and secondary clinical services are integrated into a centralized patient electronic health record (EHR), were prioritized. A feasibility analysis was conducted to identify IHSs that met the minimum requirements and allowed for close-to simultaneous introduction of CONQUEST across their primary care networks (Table 1). Feasibility criteria were informed by published literature (Powell *et al.*, 2015), the minimum requirements checklist, discussions with representatives from CONQUEST steering committees, primary and secondary care clinicians, and the DARTnet Institute, a partner organization in US QIP delivery and pivotal in data management and IHS recruitment.

**Table 1.** Five key factors considered as part of system-level feasibility assessment and their source

#	Factor(s) Assessed	Source	Components	Rationale
1	Existing Relationships & Contacts	Discussions with Stakeholders	Good Relationships and Contacts with Facilitators at the System	• It is ideal to leverage existing personal and professional networks among collaborators and/or steering committee members to facilitate initial introductions to senior executives and connect to decision-makers.
2	Primary Care Networks	Discussions with Stakeholders	Size and Availability of Primary Care Networks	• When aiming to introduce a QIP across multiple locations, it is ideal to find an integrated healthcare system with a large network of primary care practices • Working with large primary care networks also improves chances of engaging the target patient population, particularly if focusing on patients with high-risk disease, where patient groups are often smaller.
3	Health Information Exchange System + Technology	Minimum Requirements Checklist (Infrastructure + Resources)	Electronic Health Record System in Use	• To streamline data integration and exchange it is ideal to recruit integrated healthcare systems that use the same EHR platform across facilities.
		Discussions with Stakeholders	Use of Technology (e.g., e-Consults; Clinical Decision Support Systems) within existing workflows	• Assess technology use to gauge system familiarity with using similar tools to those to be adopted as part of the QIP.
		Minimum Requirements Checklist (Infrastructure + Resources)	Centralized Clinical Data Management	• When integrating data across primary care and secondary care networks, it is ideal to work with systems that have centralized data integration data-sharing processes in place
4	Prior Experience with Other Initiatives	Discussions with Stakeholders	Experience with (Sponsoring or Participating in) Quality Improvement	• Investigate prior experience to evaluate the extent of infrastructure already in place to support the delivery of QIPs, including existing Quality Improvement departments or oversight committees.
5	Interest + Capacity to Participate	Discussions with Stakeholders	Staff, Time, and Resources Available for Programme Delivery	• Interest and buy-in from senior executives are essential. • When assessing internal capacity, it is important to consider how temporal factors may impact ability to participate, such as system restructuring, changing EHR systems, high vacancy rate, natural disasters (e.g., hurricanes, wildfires), or a global pandemic.

Abbreviations: EHR, electronic health records; QIP, quality improvement programme.

The following criteria were identified as the top five factors to assess when reviewing IHS suitability alongside the rationale considered for each decision and the outcome (Table 1):

1. Existing relationships and contacts – Utilizing existing relationships can facilitate information gathering to assess feasibility and lead to introductions of key senior executives and leadership in primary care (Bonawitz *et al.*, 2020). When developing a short list of potential IHSs for CONQUEST, the QIP’s steering committee members were invited to reach out to their IHS-affiliated contacts to gauge interest. A shortlist of potential IHSs was developed based on committee recommendations and feedback from their contacts.
2. Primary care networks – In order to introduce CONQUEST across primary care practices within the desired timeline, it was important to assess IHS primary care networks. We use the term ‘primary care network’ broadly to refer to an existing infrastructure defining leadership roles and relationships among primary care practices within an IHS. For assessed IHSs, primary care practices were organized by departments, each with several practice locations, e.g., departments of family medicine, internal medicine, geriatrics, etc. Approval to introduce CONQUEST was sought from department heads, rather than administrators in individual practices. In these complex systems, leadership was highly structured; however, navigating the structure was not always entirely transparent in part due to departmental siloing.
3. Health information and exchange (HIE) systems – Use of EHR software, such as Epic or Cerner, has increased (Jiang *et al.*, 2023); between 2008 and 2021 from 9% to 96% among hospitals and from 17% to 78% among office-based physician practices (*Adoption of Electronic Health Records by Hospital Service Type 2019–2021*, 2022). As EHR adoption expands, it creates the opportunity to consolidate patient records from various care settings, allowing patients and providers access to comprehensive records to inform decision-making. This process of integrating EHRs across facilities and exchanging EHRs with third parties requires HIE systems; therefore, IHSs with established HIE systems were selected to streamline data collection and sharing for CONQUEST.
4. Experience with new initiatives – Clear organizational structure and governance of QI is related to successful QIP implementation (Dixon-Woods, McNicol and Martin, 2012). Previous experience provides IHSs with opportunities to develop and formalize procedures for reviewing and implementing QIPs. Lines of communication between departments can be established, and oversight responsibilities can be assigned, providing a clear path for new initiatives. Additionally, insight gained from past failures may prevent future mistakes, increasing the likelihood of success (Dixon-Woods, McNicol and Martin, 2012). Consequently, IHSs with previous experience in introducing new healthcare initiatives were prioritized.
5. Interest and capacity to participate – Without interest among senior management, the success of a QIP is unlikely (Sweeney *et al.*, 2018). Therefore, identifying IHSs with priorities that align with QIP aims, that support buy-in, and that have a willingness to commit can help overcome challenges during implementation (Dixon-Woods, McNicol and Martin, 2012). However, even if all conditions are met and interest is high, an IHS must also have the capacity to participate. Here, capacity refers to the extent to which an IHS possesses the necessary resources for implementation within the desired timeframe. Factors that can influence capacity include disruptive events such as switching EHR software, system restructuring, experiencing a recent natural disaster, e.g., a hurricane or wildfire, or – as was the case while recruiting IHSs for CONQUEST – experiencing a global pandemic.

The findings of the feasibility assessment are detailed in S-Table 3. We successfully recruited an IHS in Ohio, one in New York, and one in Colorado. These IHSs met all the criteria outlined in Table 1 and were determined to offer the best opportunity for CONQUEST to succeed.

## Stage 2: IHS recruitment & preparing for implementation

Launching a QIP to improve the management of patients with chronic conditions requires collaboration among multiple departments in an IHS. With CONQUEST, introducing and gaining approval for the programme, exchanging EHR data, and implementing the QIP across primary care networks required comprehensive preparation and coordination. This included the following: 1) engaging local clinicians to champion the programme to leadership and throughout primary care departments, 2) establishing comprehensive HIE and data management strategies, and 3) identifying patients meeting modifiable high-risk (MHR) criteria and securing mechanisms for patient outreach and CDS delivery (Table 2).

**Table 2.** Processes involved in preparing systems for CONQUEST implementation

Process	Stages	Ohio System	Colorado System	New York System
Recruiting Local Clinical Champions	OPC Preparation	Reviewed participation in previous Quality Improvement (QI) programmes and engaged local champions in the system.		
	Local Champions	Pulmonologist	Family Physician	Pulmonologist and Primary Care Physician
	Lesson(s)	Collaborating with champions is critical in identifying key decision-makers involved in securing approval for QI within each system and facilitates open inter-departmental communication.		
Data Acquisition for Quality Improvement	OPC Preparation	Prepared a HIPAA-compliant data hosting environment. Reviewed institutional policies for releasing data for QI purposes. Executed data use agreements and secured additional approvals from the system’s Information Technology and/or Data Management departments.		
	Local Data Management	Center for Clinical Informatics	Multi-institutional collaborative health data warehouse	Central health system data warehouse Data Use Committee
	Lesson(s)	Verifying that all contracts, protocols, and data hosting environments adhere to HIPAA compliance best practices before seeking data access reduces delays as local approval bodies confirm regulatory compliance and data security requirements.		
Data Processing	OPC Preparation	Developed clinical data management plan to standardize data across systems. The CONQUEST Database is structured according to the OMOP common data model (CDM), which standardizes health data across different healthcare systems and coding vocabularies. Obtained and organized a comprehensive library of EHR coding vocabulary to ensure all data elements are captured.		
	Local Data Extractions	Ohio data were not in the OMOP CDM and therefore required processing to standardize	Data in both systems were in the OMOP CDM; however, each system utilized Epic’s customization options, which generate additional codes that required processing to standardize	
	Lesson(s)	Establishing a clinical data management plan to standardize data is key for consistency of QI delivery, interpretation of QI data analytics, facilitation of programme expansion, and data sharing. When developing the QI timeline, allow at least one month to complete data mapping and data processing.		
Primary Care Engagement	OPC Preparation	Collaborated with local champions to map the system’s primary care network and decide which departments to include in the QI programme. Developed a presentation of the core concepts of the CONQUEST programme and personalized the materials for each department. Invited local champions to present the CONQUEST programme to primary care leadership to encourage participation.		
	Local Primary Care Departments	All practices in the departments of Internal Medicine, Family Medicine, and Medicine/Paediatrics were included. Personal emails from the local champion were sent to department leadership.	All but 2 practices in the departments of Primary Care, Family Medicine, and Internal Medicine were included. Local champion introduced primary care leadership to CONQUEST first during departmental meetings, and then in direct emails which proved more successful.	All practices in the departments of Medicine and Geriatric were included. Local champions presented CONQUEST to primary care leadership in New York.
	Lesson(s)	Encouraging 1:1 engagement between local champions and primary care leadership helps secure buy-in to QI programmes. Messaging should be concise, highlight the QI programme’s ability to support physicians, and end with a call to action to either opt-in or opt-out.		
Identifying the Modifiable High-risk Populations	OPC Preparation	Applied CONQUEST-specific algorithms to EHR data. Conducted medical review of EHR data to validate exacerbations, assess comorbidities, and review medications to ensure accurate CDS delivery and support additional algorithm refinement.		
	Local Data Availability	Ohio system ingested discrete data for spirometry results, allowing for fuller utilization of algorithm parameters.	Limited discrete spirometry data available in extracted data.	Limited discrete spirometry data available in extracted data.
	Lesson(s)	Developing an iterative process of algorithm refinement helps ensure all code vocabularies are capturing the correct data elements for each healthcare system. Engaging medically trained personnel throughout to conduct EHR reviews of identified patients ensures appropriate patients receive CDS and helps improve sensitivity and specificity of the algorithm.		
Delivering CONQUEST Questionnaires	OPC Preparation	Investigated local patient attitudes towards receiving communication from their healthcare system to inform patient outreach plans (digital, telephone). Collaborated with Information Technology services to create QI-specific telephone numbers and email addresses. Developed a project management and patient-reported information platform to track patient engagement and collect questionnaire data		
	Local Methods of Patient Outreach	Invitations were delivered via email, telephone, Epic patient portals, and the local champion coordinated the use of third-party applications for automated SMS messaging.	Digital methods of outreach required approval from a digital patient experience group. Invitations were delivered via email, telephone, and Epic patient portals.	Invitations were delivered via email and telephone.
	Lesson(s)	Prioritize the development of patient-centred engagement approaches. Engage patients in the initial stages of planning implementation, either through individual sessions or focus groups, to gather input and make necessary adjustments in outreach strategies. Employing third-party software can optimize communication efforts and expand the potential to connect with patients.		

Abbreviations: CDS: clinical decision support; CONQUEST: The COllaboratioN on QUality improvement initiative for achieving Excellence in STandards of COPD care; EHR: electronic health record; HIPAA: Health Insurance Portability and Accountability Act; OMOP: Observational Medical Outcomes Partnership; OPC: Optimum Patient Care; QI: quality improvement; SMS, short message service.

### Recruiting local clinical champions & outreach

The involvement of clinical champions played a significant role in securing IHS participation in CONQUEST. Clinical champions are individuals who are dedicated to a new initiative and embedded in clinical settings (Bonawitz *et al.*, 2020; Morena, Gaias and Larkin, 2022). They are effective in promoting the implementation of new initiatives such as QI, and they have valuable knowledge of system structure, current priorities, policy, and any local disruptions that may influence interest in participating (Wood *et al.*, 2020). The pivotal role played by CONQUEST clinical champions began with the identification of key decision-makers and relevant governing bodies. Champions then supported open and direct communication between OPC and IHS leadership throughout the approval processes (Table 2).

In advance of meeting with primary care leadership (i.e., medical directors, department heads), OPC developed information packets including CONQUEST overview material and publications. Deciding who would receive the information, how it would be delivered, and the key messages to highlight was informed by published literature (Powell *et al.*, 2015) and determined through collaboration with the local clinical champions. Methods of introducing the QIP included presentations by OPC to primary care groups and personal outreach to leadership by the clinical champion. Direct messages from the clinical champions, which proved to be the most successful approach for promoting participation, contained a concise overview of the programme that emphasized its purpose of easing heavy workloads by assisting in the management of patients with COPD. The conclusion included a clear call to action, asking recipients to respond if they preferred their department not to take part or if they desired more information.

Insights from clinical champions regarding the preferred modes of communication among department heads (e.g., whether a quick phone call was preferred to an email or a video chat was preferred over an Epic inbox message) aided in building collaborative relationships. In the dense, multimodal healthcare communication environment and with the concern of alert fatigue (*Alert Fatigue*, 2019), the method of communication proved important to consider.

### Data strategy and management

HIE is a core component of CONQUEST. The flow of data began with an IHS sharing data with OPC analysts who then applied the CONQUEST algorithms to identify MHR patients. The list of patients and EHR relevant to CDS delivery – including data related to patients’ most recent exacerbation – was shared back to the local CONQUEST delivery teams. The following tasks were carried out, many in parallel, to make this possible: 1) ethics submission and exemption review, 2) building a Health Insurance Portability and Accountability Act (HIPAA)-compliant data hosting environment, 3) executing data use agreements (DUAs) and obtaining other data-related approvals, and 4) developing a common data model (CDM) that can be used across participating IHSs (Table 2).

#### Ethics submission and exemption review

QIPs aim to systematically improve outcomes for patients, healthcare systems, and organizations (Batalden and Davidoff, 2007). As such, they do not typically meet the US Department of Health and Human Services definition of research (Office for Human Research Protections, 2016). However, it is best practice to submit QIP protocols to local regulatory oversight offices, including institutional review boards (IRBs) or Quality Leadership to obtain exempt status. Determining whether a QIP meets exemption criteria is not always straightforward; many initiatives include elements that do meet the definition of research, and it is not uncommon for QI to expand into research projects (Bass and Maloy, 2020). Although many QIPs may be exempt, a growing number of medical journals require documentation of exempt status when submitting a publication for peer review. As CONQUEST involves robust data collection and exchange, measurement of many clinical endpoints, and publications, IRB approval was sought from a central IRB, and then from each IHS.

#### HIPAA-compliant environment

To avoid delays in executing DUAs, a secure data hosting environment was first established and all data safety and security measures in OPC’s standard operating procedures manual were reviewed and updated. This included the verification of HIPAA privacy training for all employees involved with CONQUEST, documentation of security measures and technical safeguards, planning ongoing security monitoring, and thorough testing of the data hosting environment. Documents were readied to share as part of the DUA process and with the IHS’s data management groups. The experiences with CONQUEST suggest that early adoption of HIPAA guidelines and documentation of technical processes, even for QIPs sharing fully de-identified data, can help expedite the DUA process.

#### DUAs *and other approvals*

Formulating a comprehensive data strategy involved establishing the specific terms and conditions for utilizing EHR data in DUAs. In the process of coordinating the logistics of data transfers, the CONQUEST team learned of additional approvals required from IHS data management and governance groups. In one IHS, consultation and approval from a Data Use Committee overseeing all data-sharing requests was required. Additionally, two IHS data management groups required separate approval of the protocol, along with a comprehensive assessment of the CONQUEST data hosting environment.

Failing to obtain approval from data management groups would have resulted in excluding an IHS from CONQUEST, regardless of signed DUAs. The additional approval steps caused a delay, but the process provided an important learning opportunity. It highlighted the significance of developing a comprehensive data strategy in advance and working with champions to establish direct lines of communication with data management groups early in the IHS recruitment process.

#### Developing a CDM

Standardization across system databases through adoption of a CDM allows for consistency in the interpretation of QI data analytics and CDS delivery and facilitates future expansion of the QIP (Hallinan *et al.*, 2024). The CONQUEST database was structured according to the Observational Medical Outcomes Partnership (OMOP) CDM, which standardizes health data across different healthcare systems and coding vocabularies. A comprehensive library of EHR coding vocabularies (e.g., ICD-10, CPT4) was developed to ensure all data elements were captured. Although data from each IHS still required processing prior to applying the CONQUEST algorithms, adopting an established, well-known CDM such as OMOP accelerated the next step of identifying patients meeting the MHR criteria.

### CONQUEST case identification & CDS delivery

#### Case identification & algorithm refinement

MHR patients were identified by applying CONQUEST algorithms to practice EHR data. CONQUEST steering committees provided extensive input on the case identification algorithms while refinement was ongoing through an iterative review process. Clinical review of MHR patient records offered opportunities to improve the specificity of patient cohort and exacerbation identification algorithms. Review included verifying eligibility criteria and provided insight into local EHR coding practices and an understanding of IHSs’ routine care workflows. Refinements included improved accuracy in detection of COPD diagnosis and enhanced recognition of non-respiratory antibiotic- or steroid-prescribing events (e.g., urinary tract or skin infections and musculoskeletal problems).

#### CDS: establishing means of delivery

Documenting CDS activities is a crucial component of CONQUEST and provides clinicians with a more complete picture of the patient’s ongoing management (Pullen *et al.*, 2021). During the QIP development stage, the intention was to integrate CONQUEST CDS into the IHSs’ Epic EHR software. Epic allows users to customize many features. However, discussions with local CONQUEST teams revealed that integrating CDS into Epic within the desired timeframe would be unlikely due to the complexity of the customization and backlog of projects in the queue for the three IHSs’ Epic administrative teams. As a solution, the CONQUEST clinicians proposed creating CONQUEST Epic note templates to ensure that all aspects of the CDS were accounted for and recorded during patient consultations. CDS components were added to the template using Epic EHR documentation tools, e.g., key phrases and symbols that automatically populate a visit note with relevant information, allowing for consistency in CDS delivery and documentation. The solution offered by the CONQUEST clinicians serves as a reminder of the positive impact of encouraging creative problem-solving and remaining open to feedback, ultimately leading to improvements in QI delivery.

Methods of patient outreach were also established. Preparing for patient outreach involved review of relevant literature and gathering insights from local QI teams and patients to understand their communication preferences with the healthcare system. Opinions varied across IHS locations; at one IHS, patients expressed frustration due to receiving excessive calls, mainly automated reminders. Additionally, the IHS had recently changed their system so that all calls from the IHS show on caller ID as the same number, rather than from their PCP, for example. This concealed details patients previously used to decide whether to answer. In a different system, patient response varied significantly; patients shared that they were much more inclined to pick up calls from their IHS. This IHS has a centre dedicated to evaluating digital patient communications in part to minimize message exhaustion among patients. Feedback from patients regarding the experience of completing the CONQUEST questionnaires, which are part of the CDS, was also sought; the questionnaire layout and flow were amended to improve the patient experience. Following feedback, local teams established patient communication channels, such as designated CONQUEST phone lines and email addresses, authorization to communicate through patient portals, and utilization of text messaging. As demonstrated in the recruitment of primary care networks, the ability to adjust communication methods to align with the preferences of the target audience proved important for successful communication.

## Stage 3: implementation delivery models in three IHSs

### Building and supporting local QI delivery teams

As previously noted, CONQUEST was developed with a high level of flexibility. Initial implementation models were tailored for practice-driven delivery, whereby each practice would engage in patient outreach and arrange appointments with practice PCPs. After receiving feedback from the clinical champions, informed by their discussions with PCPs, these approaches were ruled out due to concerns that the QIP could be challenging for resource-strained practices and those still recovering from the height of the COVID-19 pandemic. Consequently, the implementation model was adapted to a more centralized coordination of CDS delivery within all three IHSs.

Local clinical champions assisted in identifying delivery team members, with each comprised of patient outreach coordinator(s) and central provider(s). Local teams received education in the principles of QI and underwent a comprehensive orientation to the programme with OPC prior to implementation and then continued to meet weekly with OPC to review or raise questions. Central CONQUEST clinicians were provided CDS containing considerations for assessing, treating, and managing patients based on current COPD guidelines and expert input, which was periodically updated based on clinician feedback. Inviting MHR patients to complete CONQUEST questionnaires was the first step in CDS delivery. For already diagnosed patients who were interested, an appointment with the CONQUEST central clinician was scheduled. For undiagnosed patients, spirometry was arranged. Patient care and treatment plans were customized based on individual needs and circumstances in collaboration with the patient and their PCPs, who were kept informed by the CONQUEST central provider.

Forming a central QIP delivery team within each IHS required detailed workflow development. Workflows outlined the steps for delivering each CDS element and answered key questions such as: ‘How will this step be achieved?’, i.e., how will patients complete CONQUEST questionnaires, attend consultations, or access spirometry; and ‘Who can deliver this step?’. CONQUEST CDS includes assessments and interventions that require appropriately licensed providers to deliver, e.g., ordering lab work, spirometry, or CT scans, and prescribing medications. Therefore, in the development of the workflows it was important to understand the scope of practice laws for healthcare providers in each state, e.g., clinical pharmacists and nurse practitioners, as well as any IHS-specific clinical agreements between PCPs and other healthcare providers.

**Figure 2.**
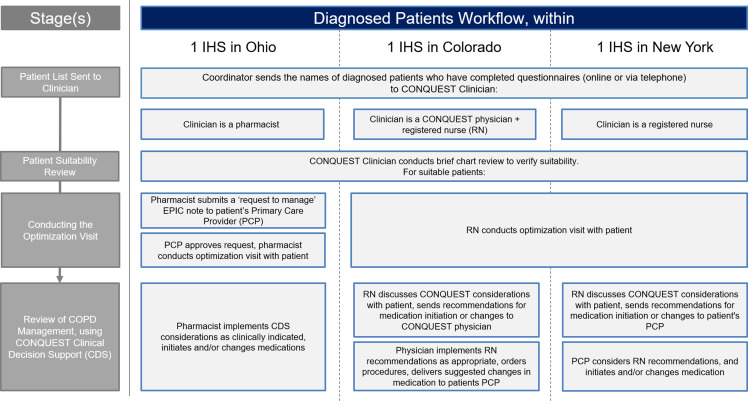
Combined locality CDS implementation strategy: diagnosed patients. Abbreviations: COPD, Chronic obstructive pulmonary disease; CONQUEST, COllaboratioN on QUality improvement initiative for achieving Excellence in STandards of COPD care; CDS, clinical decision support; IHS, integrated healthcare system; MD, medical doctor; PCP, primary care provider; RN, registered nurse.

**Figure 3.**
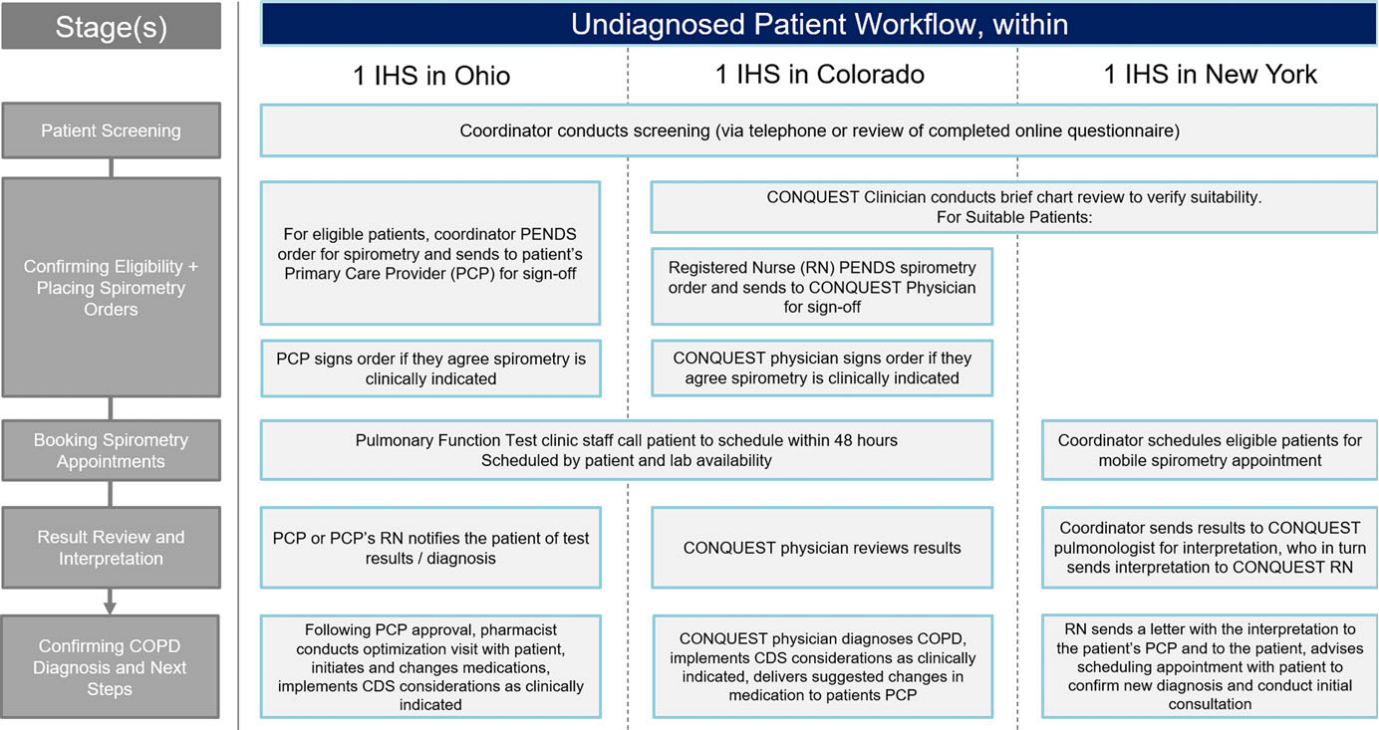
Combined locality CDS implementation strategy: potential undiagnosed COPD patients. Abbreviations: COPD, Chronic obstructive pulmonary disease; CONQUEST, COllaboratioN on QUality improvement initiative for achieving Excellence in STandards of COPD care; CDS, clinical decision support; IHS, integrated healthcare system; PCP, primary care provider; RN, registered nurse.

The three IHS implementation models are summarized below and in Figures 2 and 3.

#### Ohio

In the Ohio IHS, the CONQUEST central provider was a pharmacist with special interest in respiratory conditions. In this IHS, there was an agreement between pharmacy and primary care that allowed pharmacists to co-manage certain conditions, including COPD, following a referral from a physician. With the assistance of a CONQUEST coordinator, diagnosed patients were scheduled directly with the pharmacist. Undiagnosed CONQUEST patients who met MHR criteria were considered for spirometry. To facilitate this process, the CONQUEST coordinator submitted ‘pended’ orders, recommending spirometry to the patient’s PCP, who was notified that the patient was participating in CONQUEST and that, if diagnosed, the patient could be referred to meet with the CONQUEST pharmacist. This process was well received, with PCPs accepting the order for most of the patients referred. Throughout the process, PCPs were encouraged to schedule electronic consultations (e-consults) with a pulmonary specialist if assistance was required, particularly for cases that did not clearly meet criteria for COPD.

#### Colorado

Within the Colorado IHS, a family physician collaborated with a registered nurse (RN) in the delivery of CONQUEST CDS. With the help of the CONQUEST coordinator, the RN arranged patient visits and performed CONQUEST evaluations, providing CDS interventions that aligned with the scope of practice for RNs in Colorado. Additionally, the RN reviewed medication options with the patient and delivered recommendations to the family physician. This included suggesting spirometry for those who were undiagnosed. The family physician reviewed the recommendations, placed procedure orders, and delivered a summary of activity along with suggested changes in medication to the patient’s PCP as appropriate. This process was well received, with PCPs changing prescriptions in line with the family physician’s recommendations.

#### New York

In New York, two RNs filled the role of CONQUEST central providers. As in Colorado, the RNs provided CDS interventions that fell within their scope of practice in New York and discussed medication options with diagnosed patients. The RN then delivered considerations for medication management and interventions outside their scope to the patient’s PCP. The New York team was able to offer mobile spirometry to undiagnosed patients meeting MHR criteria and diagnosed patients who did not have spirometry recorded. Results from the mobile spirometry were reviewed by a physician; the RNs then shared the interpretation with the patient’s PCP, along with CDS considerations for next steps. For those with results indicating COPD, the RN offered to conduct future follow-up appointments.

## Discussion

This paper provides a review of the development of the CONQUEST QIP, identification and recruitment of IHSs, and implementation in three primary care networks in the United States. Throughout the three stages described, we encountered many learning opportunities that yielded valuable insights into strategies that were found to be the most beneficial. By sharing these insights, important lessons learned, and key strategies, we aim to contribute to the broader understanding of QI initiatives and their impact on driving change within healthcare systems.

Performing structured feasibility assessments of IHSs was found to be a highly efficient and effective strategy. Beyond identifying suitable IHSs, assessment tables were frequently referenced when developing recruitment materials and preparing required documents such as DUAs. Although all evaluative factors played an important role in assessing IHS recruitment potential, the decision to participate relied primarily on two key elements: leadership interest and the system’s capacity. Thus, we focused on emphasizing the alignment between CONQUEST and IHS initiatives to boost leadership interest, which proved effective. Our observation supports previous QI literature reporting leadership buy-in and aligning QI efforts with institutional goals is essential in successfully implementing QIPs (Sweeney *et al.*, 2018; Hespe, Brown and Rychetnik, 2022; Kiran, Rozmovits and O’Brien, 2023). Considering the significance of leadership interest in successful recruitment, the feasibility assessment table was updated by repositioning ‘Interest + Capacity to Participate’ from position five to position two and separating into independent factors (S-Table 2).

Highlighting CONQUEST’s alignment with IHS initiatives increased interest among leadership; however, it only marginally eased worries about capacity and the perceived burden of any new programme, irrespective of actual workload. The main concerns stemmed from ongoing staffing shortages, clinician burnout, and higher turnover following COVID-19, which is consistent with previous QIP research (Kelly *et al.*, 2022; McHugh *et al.*, 2023). The strategy that ultimately resolved capacity-related concerns was adopting centralized implementation delivery models developed with the assistance of clinical champions. However, there were limitations to this approach. The differences in practice scope between central providers in the three IHSs (Table 2) resulted in different patient pathways to optimized treatment. Operationally, this made monitoring progress in a centralized dashboard challenging as each IHS had different patient touchpoints.

The strategic recruitment of clinical champions connected to primary care networks and IHS leadership was critical. They not only helped tailor implementation models to fit available resources and recruit central delivery team members but also assisted in navigating the approval process and secured support from primary care department leadership. Their effectiveness was in part driven by their ability to overcome departmental silos, a known barrier to QI implementation (Bonawitz *et al.*, 2020; Alexander *et al.*, 2022; Hespe, Brown and Rychetnik, 2022), and push processes forward when delays occurred.

Clinical champions direct outreach to decision-makers through their preferred channels, e.g., EPIC inbox, email, or messaging apps, with clear and action-focused messages proved more effective than formal presentations to department leadership. This aligns with recent research on recruitment strategies, which emphasizes the importance of personal communication and leveraging existing relationships for successful practice recruitment (Buckley, McHugh and Riordan, 2023). Considering our observation that the method of message delivery played an important role in moving the programme forward, future studies may benefit from considering digital message delivery channels in the evaluation of QI recruitment strategies.

Facilitating regular team meetings was key to fostering collaboration within the central delivery team. Attendance of all team members was expected, including clinical champions, central providers, clinical directors, patient outreach coordinators, and programme managers. By establishing recurring meetings, sending reminders with key agenda topics, and encouraging participation, we worked to create a space where everyone could voice their questions and concerns. This accelerated decision-making when team members reported potential issues with the implementation workflow, allowing modifications to be made to address concerns while ensuring the core elements of the programme were delivered effectively. Our experience supports conclusions from previous studies finding that fostering partnerships between team leaders and those on the ‘front line’ of service delivery is critical to ensuring consistent QI delivery (Hill *et al.*, 2020; Alexander *et al.*, 2022).

Employing strategic data management processes allowed us to circumvent top-level data access barriers, particularly those related to EHR data extraction and transitions between EHR systems (McHugh *et al.*, 2023; Carbonell *et al.*, 2024). However, establishing data sharing is a complex process and may present administrative challenges (Carbonell *et al.*, 2024; Cascini *et al.*, 2024). In this instance, the challenge arose from unanticipated requirements for additional approvals from governing bodies not identified during the assessment phase. Therefore, it is advisable to seek guidance from those who have gone through the process as part of assessing an IHS’s prior experience with QI (Table 1). Further, it is important to establish a database structure in advance, particularly when exchanging data across multiple IHSs (Hallinan *et al.*, 2024). Adopting the OMOP CDM for the CONQUEST database allowed for consistency in data analysis and algorithm refinement leading to increased specificity in identifying MHR patients across the IHSs (Hallinan *et al.*, 2024). Including spirometry results in the identification algorithms would have further improved case identification. However, not all participating IHSs were able to include results in their data and had this been a requirement in the feasibility assessment, the number of suitable IHS would have been significantly limited. Equally, although it was unfortunate that we could not fully integrate CDS elements into IHS’s Epic EHR, the solution of building the Epic note templates proved to be an effective compromise.

### Limitations

As this is not a formal evaluation of implementation processes, we are unable to make any definitive claims as to which processes proved to be the most effective. Rather, our aim is to provide a review of our experiences and offer insight to others interested in introducing QIPs for chronic disease management in primary care settings.

## Conclusion

The CONQUEST QIP aims to improve the identification, clinical evaluation, treatment optimization, and ongoing management of patients with COPD. In this paper, we outline the progression of CONQUEST’s development and implementation with the goal of contributing to the literature on QI and providing information and strategies to others interested in implementing QIPs for chronic conditions in primary care settings.

Our three-stage process includes the steps taken to formalize the CONQUEST QIP and CDS within the GOP and the criteria essential for evaluating the suitability of IHSs, providing the rationale for each. This includes an assessment of existing relationships, primary care networks, HIE systems, interest among leadership, capacity for participation, and experience with QI. A template highlighting these factors is available for those interested in conducting their own assessments. Stage two also provides a description of the processes involved in preparing IHSs for implementation. These include recruiting local clinical champions, establishing a data management framework, refining patient identification algorithms, and preparing CDS delivery and patient outreach. Stage three is the evaluation of implementation models used by healthcare systems.

Throughout, we present insights derived from our experiences, highlighting the strategies that demonstrated the greatest effectiveness. Key strategies included conducting a structured feasibility assessment and emphasizing the alignment of CONQUEST QIP with IHS initiatives to foster interest from leadership during recruitment. We also discuss how flexibility with implementation models can mitigate leadership concerns. Additionally, facilitating team meetings and creating a collaborative environment was beneficial and allowed for quick decision-making.

Learnings from this development paper also inform recommendations for future research, including investigating the ongoing impact of COVID-19 on QI participation and the effectiveness of various digital communication methods in inter/intra-departmental correspondence. More broadly, the literature would benefit from additional development papers detailing processes in QIP development and implementation, and exploration of factors critical to assess when considering QI recruitment in other healthcare settings.

## Supporting information

Evans et al. supplementary material Evans et al. supplementary material

## Supplementary material

The supplementary material for this article can be found at https://doi.org/10.1017/S1463423625100170
