# Supplementary material for: Practical strategies for achieving system change in the US: lessons and insights from the CONQUEST quality improvement programme
Source: Prim Health Care Res Dev. 2025 Jun 23;26:e50. doi: 10.1017/S1463423625100170 (PMC12188133; doi:10.1017/S1463423625100170)
Supplement: Evans et al. supplementary material [file S1463423625100170sup001.docx]

# Supplemental

**S-table1. Definitions of Key Terms (Adapted from Pullen et al. 2021)**

| Term | Definition |
| --- | --- |
| Target Population | |
| Patients with Modifiable High-risk Chronic Obstructive Pulmonary Disease (COPD) | Patients with COPD (or potential COPD) who have experienced ≥2 moderate, or ≥1 severe exacerbations in the last 24 months (with at least one exacerbation occurring in the last 12 months), AND whose medical record data indicates clearly that there is scope for management optimization. |
| Patients with Scope for Management Optimization | Patients who continue to exacerbate while on their current therapy (including no therapy, SABA, SAMA or SABA/SAMA only) and/or whose management may be optimized by correct diagnosis, or additional pharmacological and non-pharmacological interventions. |
| Already Diagnosed Patients with Modifiable High-risk COPD | Patients with an existing COPD diagnosis who fit the modifiable high-risk criteria with respect to recent COPD exacerbations and are ≥40 years of age, and in whom there is an opportunity to optimize management. |
| Undiagnosed Patients with Potential Modifiable High-risk COPD | Patients without a COPD diagnosis who fit the modifiable high-risk criteria with respect to recent exacerbations of potential COPD, and who are smokers or former smokers ≥40 years of age. |
| Newly Diagnosed Patients with Modifiable High-risk COPD | A subset of the above-undiagnosed patients who receive a COPD diagnosis following diagnostic assessment. |
| Exacerbations | |
| COPD exacerbation | A significant worsening in respiratory symptoms in people with COPD (or an event analogous to a COPD exacerbation in people with suspected but undiagnosed COPD), categorized as moderate or severe (see below) using validated code lists. |
| Moderate exacerbation | Requiring an acute course of systemic corticosteroids and/or a course of antibiotics within 3 days of a lower respiratory consultation indicative of a probable COPD exacerbation, or an emergency room visit for a COPD-related cause. * |
| Severe exacerbation | An exacerbation resulting in a hospital admission. * |
| Other | |
| Major Adverse Cardiac or Respiratory Event | Occurrence of any of the following events:  New diagnosis for heart failure, Hospitalization for heart failure, Coronary Artery Revascularization, Myocardial Infarction, Ischemic stroke, All-cause mortality (further categorized as sudden death, cardiac deaths, respiratory death, none of these), Hospitalization (admittance) for respiratory event*, or Complicated exacerbations**. |
| *Identified using validated code lists for moderate and severe COPD exacerbations.  ** Exacerbations requiring additional hospitalization or treatment with acute doses of oral corticosteroids or antibiotics between 8 and 28 days after the start of the initial exacerbation. | |
| **Abbreviations:** COPD, chronic obstructive pulmonary disease; SABA, short-acting β2-agonist; SAMA, short-acting muscarinic antagonist. | |

### S-table 2. Printable Feasibility Checklist

|  | Factors Assessed | Components | Criteria Met? |
| --- | --- | --- | --- |
| 1 | Relationships & Contacts | Good Relationships and Contacts with Key Leaders at the System |  |
|  |  | Presence of Quality Lead/Team at the System |  |
|  |  | Ability to Conduct Face-to-face and Teleconference Meetings with System Representatives |  |
| 2 | Interest and Alignment with Current Initiatives | Existing or Planned Initiatives which Align with Program Objectives |  |
| 3 | Capacity to Participate | Staff, Time and Resources Available for Program Delivery |  |
| 4 | Primary Care Networks | Presence and Availability of Primary Care Networks |  |
| 5 | Health Information Exchange System + Technology | Electronic Health Record System in Use – consistent across participating locations to streamline data processing and delivery of CDS (e.g., sharing Epic templates) |  |
|  |  | Use of Technology (e.g. e-Consults; Clinical Decision Support Systems) |  |
|  |  | Centralized data management department/partner responsible for integrating patient data across system’s departments, locations, and affiliated clinical networks. |  |
| 6 | Prior Experience with  (Sponsoring or Participating in)  Other New Initiatives | Previous Experience with Quality Improvement |  |

**S-table 3. Outcomes of US Healthcare System Feasibility Assessment**

| System # | | 1 | 2 | 3 | 4 | 5 | 6 | 7 | 8 |
| --- | --- | --- | --- | --- | --- | --- | --- | --- | --- |
|  | Primary System  Location (State) | North Carolina | **Colorado** | California | South-eastern US (Various) | Alabama | Michigan | **Ohio** | **New York** |
| Factor Assessed | |  |  |  |  |  |  |  |  |
| 1 | **Relationships + Contacts** |  |  |  |  |  |  |  |  |
|  | Contacts with Key Leaders / Local Clinical Champions | ✓ | **✓** | ✓ | ✓ | ✓ | ✓ | **✓** | **✓** |
|  | Existing Quality Improvement/ Team Available | ✓ | **✓** | ✓ | ✓ | ✓ | ✓ | **✓** | **✓** |
| 2 | **Size of Primary and  Secondary Care Network** |  |  | X |  | X |  |  |  |
|  | # of Primary Care Locations | 65 | **85** | 40 | 200+ | 11 | 150+ | **60+** | **30+** |
|  | # of Hospitals | 10 | **13** | 4 | 40+ | 3 | 3 | **4** | **8** |
|  | Estimated COPD Population | 36,500 | **47,000** | 13,400 | 20,000 | - | - | **20,000** | **20,000** |
| 3 | **Health Information Exchange/ System Technology** |  |  | X | X | X |  |  |  |
|  | Data Access for Sponsors | ✓ | **✓** | X | ✓ | ✓ | ✓ | **✓** | **✓** |
|  | Use of Epic Electronic Health Records | ✓ | **✓** | ✓ | X | X | ✓ | **✓** | **✓** |
|  | Data Integration Across Primary and Secondary Care | ✓ | **✓** | ✓ | ✓ | ✓ | ✓ | **✓** | **✓** |
|  | Use of Clinical Technology | ✓ | **✓** | ✓ | ✓ | ✓ | ✓ | **✓** | **✓** |
| 4 | **Prior Experience** |  |  |  |  |  |  |  |  |
|  | Experience with Quality Improvement Programs | ✓ | **✓** | ✓ | ✓ | ✓ | ✓ | **✓** | **✓** |
| 5 | **Capacity to Participate** | X |  |  |  |  | X |  |  |
|  | System Level Interest | ✓ | **✓** | ✓ | ✓ | ✓ | ✓ | **✓** | **✓** |
|  | Staff + Resource Availability | X | **✓** | ✓ | ✓ | ✓ | X | **✓** | **✓** |
